# Identification of lysophosphatidylthreonine with an aromatic fatty acid surrogate as a potent inducer of mast cell degranulation

**DOI:** 10.1016/j.bbrep.2016.09.013

**Published:** 2016-10-08

**Authors:** Takayuki Kishi, Hiroki Kawana, Misa Sayama, Kumiko Makide, Asuka Inoue, Yuko Otani, Tomohiko Ohwada, Junken Aoki

**Affiliations:** a Graduate School of Pharmaceutical Sciences, Tohoku University, 6-3, Aoba, Aramaki, Aoba-ku, Sendai 980-8578, Japan; b Graduate School of Pharmaceutical Sciences, University of Tokyo, 7-3-1, Hongo, Bunkyo-ku, Tokyo 113-0033, Japan; c PRESTO, Japan Science and Technology Agency, Japan; d AMED-CREST, Japan Agency for Medical Research and Development, Japan

**Keywords:** Mast cell, Degranulation, Receptor, Lysophosphatidylserine, Lysophospholipid

## Abstract

Upon various stimulations, mast cells (MCs) release a wide variety of chemical mediators stored in their cytoplasmic granules, which then initiates subsequent allergic reactions. Lysophosphatidylserine (LysoPS), a kind of lysophospholipid, potentiates the histamine release from MCs triggered by antigen stimulation. We previously showed through structure-activity studies of LysoPS analogs that LysoPS with a methyl group at the carbon of the serine residue, i.e., lysophosphatidylthreonine (LysoPT), is extremely potent in stimulating the MC degranulation. In this study, as our continuing study to identify more potent LysoPS analogs, we developed LysoPS analogs with fatty acid surrogates. We found that the substitution of oleic acid to an aromatic fatty acid surrogate (C3-pH-*p*-O-C11) in 2-deoxy-1-LysoPS resulted in significant increase in the ability to induce MCs degranulation compared with 2-deoxy-1-LysoPS with oleic acid. Conversion of the serine residue into the threonine residue further increased the activity of MC degranulation both in vitro and in vivo. The resulting super agonist, 2-deoxy-LysoPT with C3-pH-*p*-O-C11, will be a useful tool to elucidate the mechanisms of stimulatory effect of LysoPS on MC degranulation.

## Highlights

• Lysophosphatidylserine (LysoPS) stimulates degranulation of mast cell (MC).
• We evaluated various LysoPS analogs for their MC degranulation-stimulating activity.
• We identified a threonine containing LysoPS analogs as a super agonist.
• The MC LysoPS receptor is different from the known LysoPS receptors.
• The super agonist helps to identify the putative MC LysoPS receptor.

## 1 Introduction

Mast cells (MCs) play a critical role in immediate-type allergic reactions triggered by antigen binding–induced cross-linking of IgE-bound FcεRI (a high-affinity receptor for IgE) and the resulting release of chemical mediators such as histamine and serotonin from their secretary granules, a process known as MC degranulation [1], [2]. The released histamine from MCs can cause allergic diseases such as pollinosis, urticaria, atopic dermatitis, and asthma. Identification of factors that modulate MC degranulation would be helpful in providing tools to investigate the molecular mechanisms of allergic reactions as well as to develop anti-allergic drugs.

Lysophosphatidylserine (LysoPS; 1-acyl-2-lyso-PS or 1-lyso-2-acyl-PS) has several biological activities, including a promotion of neurite outgrowth, a suppression of T lymphocyte proliferation and an enhancement of MC degranulation [3]. Among them, the most characterized biological action of LysoPS is its action to MCs. Exogenous phosphatidylserine (PS) or LysoPS strongly enhances the degranulation of rat peritoneal MCs (RPMCs) initiated by FcεRI cross-linking [4], [5]. Because LysoPS is **∼**1000 times more active than PS and LysoPS is readily produced from PS, LysoPS is considered as the true effector for MCs [5]. The action of LysoPS is highly specific; all other lysophospholipids (including lysophosphatidyl-*D*-serine, an optical isomer of lysophosphatidyl-*L*-serine) are reported to be ineffective [6]. Thus, a specific receptor for LysoPS should exist on the plasma membrane of RPMCs.

Four G protein-coupled receptors (GPCRs) (LPS_1_/GPR34, LPS_2_/P2Y10, LPS_2L_/A630033H20Rik and LPS_3_/GPR174) that were specifically activated by LysoPS have been proposed as candidate MC LysoPS receptors [8], [9]. However, they do not appear to be MC LysoPS receptors because none of them reacted with the super agonist, lysophosphatidylthreonine (LysoPT; see below) [10]. In addition, MCs isolated from LPS_1_/GPR34-deficient mice were still activated by LysoPS [11]. Thus, the LysoPS receptor on MC has not been identified yet.

For molecular identification of unknown receptor, specific agonists with potent activity are definitely useful. We previously synthesized a series of chemically modified LysoPS (*so*-called LysoPS analogs) and tested their ability to promote antigen-induced RPMC degranulation and to activate cloned LysoPS receptors (i.e., LPS_1_/GPR34, LPS_2_/P2Y10 and LPS_3_/GPR174) [10], [12], [13]. In these studies, LysoPS was regarded as a modular assembly of serine, phosphate, glycerol and fatty acid and modified each module. Indeed, we developed several LysoPS analogs containing simple modifications in individual modules that were found to be potent inducers of MC degranulation, but were incapable of activating the cloned LysoPS receptors. In the first round of our study, modification was focused on the serine and glycerol modules. As a result, a LysoPS analog with an addition of a methyl group at the carbon of the serine residue, i.e., LysoPT, was identified as a potent LysoPS analog in promoting antigen-elicited MC degranulation [10]. In the second round, the modification was focused on the fatty acid module and a number of LysoPS analogs with fatty acid module and aromatic fatty acid modifications were generated [12], [13]. Here, a number of these analogs were evaluated for their ability to promote antigen-induced MC degranulation. We identified an aromatic fatty acid surrogate that greatly enhanced the degranulation-promoting activity of LysoPS. By introducing this surrogate into the structure of 2-deoxy-1-LysoPT, we created a super agonist with ~100-fold higher activity than LysoPS.

## 2 Methods

### 2.1 Materials

Oleoyl (18:1)-LysoPS was purchased from Avanti Polar Lipids. LysoPS analogs except 2-deoxy-1-C3-pH-*p*-O-C11-LysoPT were synthesized as described previously [10], [13], and the chemical structure was confirmed by nuclear magnetic resonance (NMR) and element analysis. We confirmed that the purity of LysoPS analogs was always more than 95%. All rats and mice were purchased from Japan SLC.

### 2.2 Mast cell degranulation assay in vitro

MCs from the peritoneal cavity of rats and mice were prepared essentially as previously described [10]. The MCs were purified by Percoll (GE Healthcare) in HEPES-buffered Tyrode (HBT) solution (pH 7.4) and suspended at a cell density of 5×10^4^/mL (in 0.2 mL) in HBT solution containing 0.01% BSA and stimulated with the indicated dose of each LysoPS analog (nM–µM) in the presence of concanavalin A (Sigma Aldrich) (100 µg/mL for RPMC and 10 µg/mL for mouse peritoneal MCs), which is known to cross-link the FcεRI receptor, at 37 °C. After 15 min, cells were added with cold 0.01% BSA/ HBT solution to stop the degranulation reaction. Then, cell suspensions were centrifuged for 5 min at ×860*g*. The histamine content in the supernatant was determined by the *o*-phthalaldehyde (OPA) fluorometric assay as shown below [10], [14]. HCl (1 M) was added to the supernatant for measuring the total histamine. Next NaOH (1 M) was added, followed by OPA reagent. After 4 min, HCl (3 M) was added to stop the OPA reaction. The solution was then transferred to black 96-well flat bottom plate (Greiner) and the fluorescence at 450 nm resulting from excitation at 360 nm was measured using a microplate reader (FlexStation3, Molecular Devices). Histamine release was calculated as a percentage of the total histamine recovered from non-stimulated cells (treated with 0.01% BSA/HBT solution). Values that are subtracted by that of negative control group are shown in Figure. Values for histamine release are presented as the means±SE for several replicate experiments on different samples of pooled cells, each in triplicate. All animal procedures were done according to the guidelines for care and use of laboratory animals approved by Tohoku University.

### 2.3 Evaluation of hypothermic effect of LysoPS analogs

LysoPS and LysoPS analogs were dissolved in PBS containing 0.1% bovine serum albumin and were injected intravenously into mice. Rectal temperatures were measured with an electronic thermometer (Physitemp Instruments) every 5 or 10 min for 70 min

### 2.4 TGFα shedding assay

Transforming growth factor α (TGFα) shedding assay was performed as previously described [9]. Briefly, HEK293A (for LPS_1_ and LPS_2_) or HEK293FT (for LPS_3_) cells were transfected with two kinds of plasmids vectors encoding LysoPS receptors and alkaline phosphatase-tagged TGFα. For LPS_1_, a plasmid vector encoding Gαq/i1 was co-transfected [12], [15]. After 24 h, cells were harvested and mixed with a test compound for 1 h. Compound-induced AP-TGFα release from cells was evaluated by determining the AP activity in the conditioned media. Values for AP-TGFα release were presented as the means±SD for three independent experiments, each in triplicate.

### 2.5 Synthesis of 2-deoxy-1-C3-pH-p-O-C11-LysoPT

2-deoxy-1-C3-pH-*p*-O-C11-LysoPT was synthesized from *L*-threonine, mono-protected propane-1, 3-diol and fatty acid surrogate (C3-pH-*p*-O-C11) in a similar manner as in [13]: mono-protected propane-1,3-diol, i.e., 3-((*tert*-butyldimethylsilyl)oxy)propan-1-ol, synthesized from propane-1,3-diol, was connected with O-phosphanyl N- and C-terminal-diprototected *L*-threonine, tert-butyl O-(tert-butoxy(diisopropylamino)phosphanyl)-N-(*tert*-butoxycarbonyl)-L-threoninate by using phosphoramidite method. After removing the protective group (a *tert*-butyldimethylsilyl group) from the alcohol moiety, the free hydroxyl group was esterified with the fatty acid surrogate (C3-pH-*p*-O-C11), to yield protected. Global deprotection with TFA (trifluoroacetic acid) and purification by silica gel column chromatography furnished the 2-deoxy-1-LysoPT analog. 2-deoxy-1-C3-pH-*p*-O-C11-LysoPT as TFA salt (0.67 TFA) satisfied elemental analysis (Purity: >99.7%). HPLC purity (area normalization method): 95%.

## 3 Results and discussion

### 3.1 LysoPS analogs with an aromatic fatty acid surrogate, C3-pH-p-O-C11, is a potent mast cell degranulation inducer

**Fig. 1:**
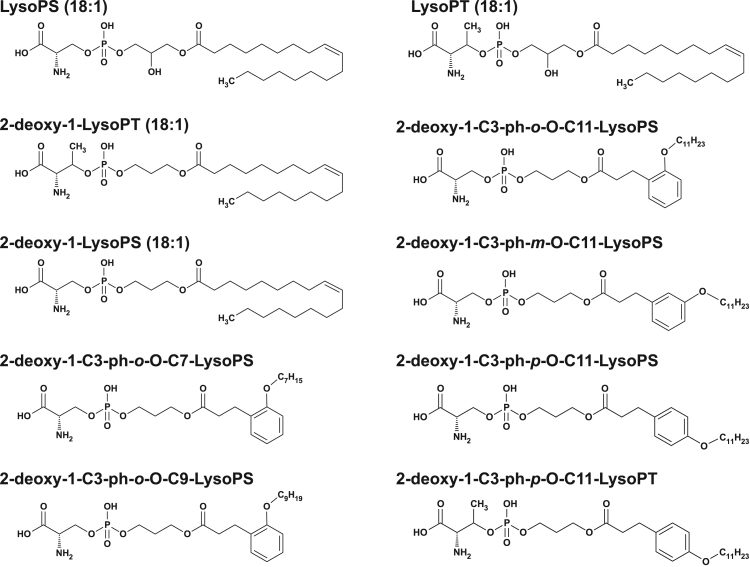
Chemical structures of LysoPS analogs used in this study.

**Fig. 2:**
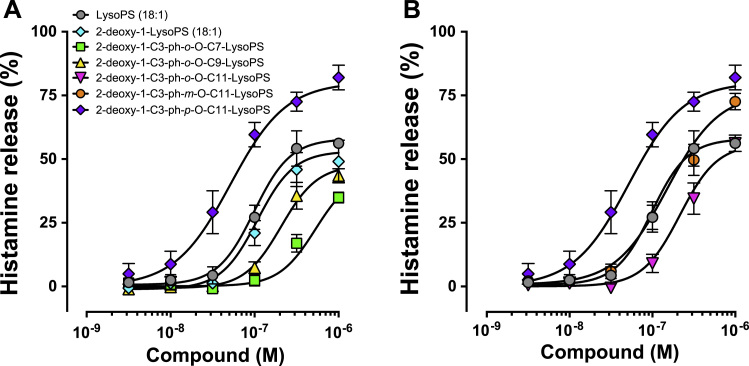
Identification of C3-pH-*p*-O-C11 as the module that confer the greatest MC degranulation activity LysoPS (18:1) and 2-deoxy-LysoPS analogs with different fatty acid surrogates (Fig. 1) were tested for their ability to induce histamine release from concanavalin A-treated RPMCs. Released histamine, expressed as a percent of the total cell histamine, was determined by fluorometric assay. Values are the means±SE of four independent experiments, each performed in duplicate.

The structure of LysoPS analogs used in this study is shown in (Fig. 1). Because of the ease of chemical synthesis and the fact that they don’t require a *sn*-2 hydroxy group of LysoPS for MC degranulation-inducing activity, most of the LysoPS analogs tested in this study did not have the *sn*-2 hydroxy group (deoxy-LysoPS analogs). We replaced the fatty acid of LysoPS with various aromatic fatty acid surrogates (Fig. 1). The LysoPS analogs tested were 2-deoxy-1-C3-pH-*o*-O-C7-lysophosphatidylserine (LysoPS), 2-deoxy-1-C3-pH-*o*-O-C9-LysoPS, 2-deoxy-1-C3-pH-*o*-O-C11-LysoPS, 2-deoxy-1-C3-pH-*m*-O-C11-LysoPS and 2-deoxy-1-C3-pH-*p*-O-C11-LysoPS. Each LysoPS analog (+ a previously characterized deoxy-1-LysoPS (18:1) and LysoPS (18:1)) was tested for their ability to stimulate concanavalin A-induced MC degranulation from RPMC. The reactivity of each LysoPS analog in stimulating MC degranulation, as judged by histamine release from RPMCs, is shown in Fig. 2. and the results are summarized in Table 1. Modification of the fatty acid module significantly affected the degranulation-stimulating activity of LysoPS. Among the 2-deoxy-1-LysoPS analogs with fatty acid surrogates, 2-deoxy-1-C3-pH-*p*-O-C11-LysoPS showed the highest activity with an EC_50_ value about 40 nM (Fig. 2 and Table 1).

**Table 1:** EC_50_ values of LysoPS analogs for rat peritoneal mast cell degranulation.

LysoPS or LysoPS analogs	EC_50_
LysoPS (18:1)	~150 nM
2-deoxy-1-LysoPS (18:1)	~200 nM
2-deoxy-1-C3-pH-*o*-O-C7-LysoPS	>500 nM
2-deoxy-1-C3-pH-*o*-O-C9-LysoPS	~300 nM
2-deoxy-1-C3-pH-*o*-O-C11-LysoPS	~300 nM
2-deoxy-1-C3-pH-*m*-O-C11-LysoPS	~100 nM
2-deoxy-1-C3-pH-*p*-O-C11-LysoPS	~40 nM

### 3.2 2-deoxy-1-C3-pH-p-O-C11-LysoPT is the most potent mast cell degranulation inducer

**Fig. 3:**
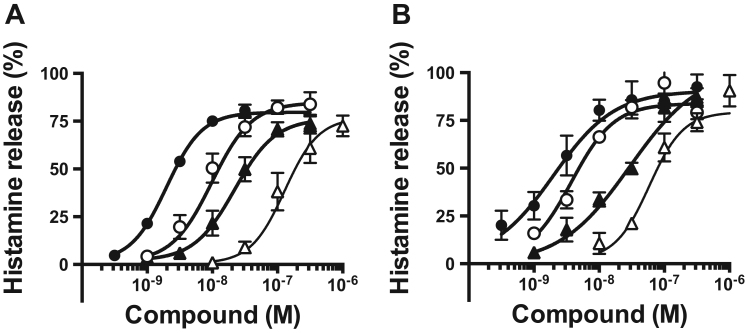
2-deoxy-1-C3-pH-*p*-O-C11-LysoPT is a super agonist for MC degranulation 2-deoxy-1-C3-pH-*p*-O-C11-LysoPT and related compounds (LysoPS (18:1), LysoPT (18:1) and 2-deoxy-1-C3-pH-*p*-O-C11-LysoPS for comparison) were tested for their ability to induce histamine release from concanavalin A-treated rat (A) and mouse (B) peritoneal MCs. Released histamine was determined by fluorometric assay and the histamine release is expressed as a percent of the total cell histamine. Values are the means±SE of three independent experiments, each in duplicate. Each symbol represents; closed circle (**•**) 2-deoxy-1-C3-pH-*p*-O-C11-LysoPT, open circle (**○**) LysoPT (18:1), closed triangle (**▲**) 2-deoxy-1-C3-pH-*p*-O-C11-LysoPS and open triangle (**△**) LysoPS (18:1).

Because conversion of serine polar head to threonine dramatically enhanced the activity to stimulate degranulation reaction of LysoPS [10], we thus synthesized 2-deoxy-1-C3-pH-*p*-O-C11-LysoPT (Fig. 1) and tested its ability to stimulate degranulation reaction (Fig. 3A and Table 2). The resulting LysoPT analog, 2-deoxy-1-C3-pH-*p*-O-C11-LysoPT, was extremely potent in stimulating degranulation from RPMCs, with EC_50_ value about 3 nM. We also confirmed that 2-deoxy-1-C3-pH-*p*-O-C11-LysoPT was the most potent for inducing degranulation of mouse peritoneal MCs with EC_50_ value about 3 nM (Fig. 3B and Table 2).

**Table 2:** EC_50_ values of LysoPS analogs for rat (RMPC) and mouse (MPMC) peritoneal mast cell degranulation.

LysoPS or LysoPS analogs	EC_50_	EC_50_
	RPMC	MPMC
LysoPS (18:1)	~150 nM	~80 nM
LysoPT (18:1)	~10 nM	~7 nM
2-deoxy-1-C3-pH-*p*-O-C11-LysoPS	~40 nM	~30 nM
2-deoxy-1-C3-pH-*p*-O-C11-LysoPT	~3 nM	~3 nM

### 3.3 In vivo hypothermic effect

**Fig. 4:**
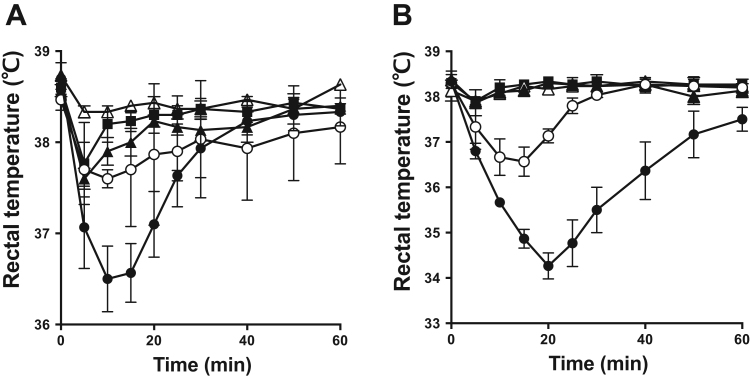
Hypothermic effect of LysoPS analogs C57BL/6 mice were injected intravenously with 5 µg (A) or 10 µg (B) of 2-deoxy-1-C3-pH-*p*-O-C11-LysoPT and related compounds (LysoPS (18:1), 2-deoxy-1-LysoPS (18:1), 2-deoxy-1-LysoPT (18:1) and 2-deoxy-1-C3-pH-*p*-O-C11-LysoPS for comparison) at indicated dosage and rectal temperature was monitored every 5 or 10 min. Data are representative of three experiments, each in triplicate. Each symbol represents; closed circle (**•**) 2-deoxy-1-C3-pH-*p*-O-C11-LysoPT, open circle (**○**) 2-deoxy-1-LysoPT (18:1), closed triangle (**▲**) 2-deoxy-1-C3-pH-*p*-O-C11-LysoPS, open triangle (**△**) 2-deoxy-1-LysoPS (18:1) and closed square (■) LysoPS (18:1).

Intravenous injection of 100 µg of LysoPS in mice induced transient hypothermia due to systemic MC degranulation [10]. We focused four LysoPS analogs and LysoPS (18:1) and tested their hypothermic effects. Mice were injected *i.v.* with the potent LysoPS analogs, and decrease in the rectal temperature was measured. Unlike LysoPS, only 5 or 10 µg *i.v.* injection of 2-deoxy-1-C3-pH-*p*-O-C11-LysoPT induced dramatic hypothermia. The rank order matched to that observed for in vitro degranulation-stimulating activity (Fig. 4). Again, 2-deoxy-1-C3-pH-*p*-O-C11-LysoPT was induced the greatest hypothermic action.

### 3.4 2-deoxy-1-C3-pH-p-O-C11-LysoPT did not activate LPS_1-3_

**Fig. 5:**
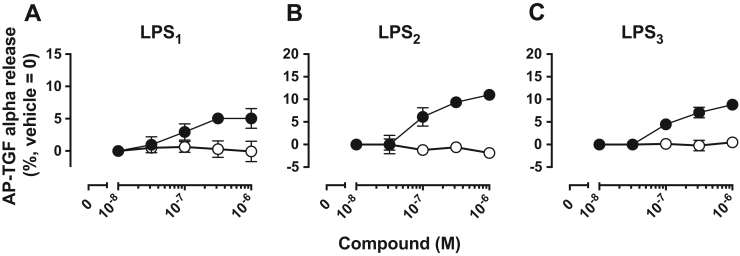
2-deoxy-1-C3-pH**-*****p***-O-C11-LysoPT did not activate LPS_1_, LPS_2_ and LPS_3_ (A, B) TGFα shedding responses of HEK293A cells expressing LPS_1_ (A) and LPS_2_ (B) induced by LysoPS (18:1) (closed circles) or 2-deoxy-1-C3-pH-*p*-O-C11-LysoPT (open circles). Data are representative of three experiments. Error bars are SD (standard deviation) for three assay replicates for one experiment. (C) TGFα shedding response of HEK293FT cells expressing LPS_3_ to LysoPS (closed circles) or 2-deoxy-1-C3-pH-*p*-O-C11-LysoPT (open circles). Data are representative of three experiments. Error bars are SD for three assay replicates for one experiment.

To examine if 2-deoxy-1-C3-pH-*p*-O-C11-LysoPT activates LPS_1–3_, we used TGFα shedding assay. While LysoPS activated all the three LysoPS receptors in TGFα shedding assay, 2-deoxy-1-C3-pH-*p*-O-C11-LysoPT didn’t (Fig. 5). These results suggest that the putative LysoPS receptor on MCs is different from the cloned GPCR-type LysoPS receptors.

Lysophosphatidylserine (LysoPS) is composed of four modules, i.e., a serine, a phosphate, a glycerol and a fatty acid, which are chemically linked by phosphodiester or ester bonds. We previously examined structure-activity relationship (SAR) of LysoPS by synthesizing a number of LysoPS analogs with modifications of the four modules, and by evaluating them for both MC degranulation and activation of the cloned GPCR-type LysoPS receptors (LPS_1_, LPS_2_ and LPS_3_) [10], [12], [13]. Initially our modification was focused exclusively on serine and glycerol modules [10]. As a result, threonine was found to be superior to serine for MC degranulation. In this study, to identify a preferable fatty acid module, we used a new set of LysoPS analogs with modification in the fatty acid module [13]. We first identified C3-pH-*p*-O-C11 as the fatty acid module that conferred the greatest MC degranulation activity (Fig. 2 and Table 1). Introducing the fatty acid surrogate into the structure of a potent ligand, 2-deoxy-1-LysoPT, made it possible to identify a super agonist, i.e., 2-deoxy-1-C3-pH-*p*-O-C11-LysoPT. Indeed, the activities of the resulting LysoPS analogs showed 100 times higher than LysoPS (18:1) both in vitro (Fig. 3 and Table 2) and in vivo (Fig. 4). The SAR for MC degranulation obtained in this study was different from the SAR for the LysoPS receptors (LPS_1_, LPS_2_ and LPS_3_) [13]. It should be noted that 2-deoxy-1-C3-pH*-o*-O-C11-LysoPS, a poor inducer of MC degranulation (Fig. 2 and Table 1), was actually a potent agonist for cloned GPCR-type LysoPS receptors [13]. We previously showed that 1-stearoyl (18:0)-LysoPS is a potent agonist for MC degranulation [10], while it was poor agonist for all the cloned GPCR-type LysoPS receptors. By contrast, LysoPS with unsaturated fatty acid such as oleic acid (18:1) and arachidonic acid (20:4) was preferable ligand for cloned GPCR-type LysoPS receptors. Thus, it is reasonable to assume that the ligand recognizing pocket of putative MC LysoPS receptor is quite different from those of cloned GPCR-type LysoPS receptors. Taking account of the fact that the structure of C3-pH-*p*-O-C11 is more linear than C3-pH-*o*-O-C11 (Fig. 1), it can be speculated the pocket of the putative MC LysoPS receptor accommodates to linear fatty acids, while those of the cloned GPCR-type LysoPS receptors accommodate to bended or curved fatty acids. We also confirmed that 2-deoxy-1-C3-pH-*p*-O-C11-LysoPT activated none of the cloned GPCR-type LysoPS receptors in TGFα shedding assay (Fig. 5), suggesting again that the ligand binding manner of MC LysoPS receptor is different from those of the cloned GPCR-type LysoPS receptors.

In summary, we have identified a potent LysoPS analog, 2-deoxy-1-C3-pH-*p*-O-C11-LysoPT, which induced MC degranulation at nM concentration. The super agonist should be useful not only for pharmacological characterization of the MC LysoPS receptor but also for biochemical identification of the receptor.
